# Potential Targets and Signaling Mechanisms of Cinnamaldehyde Enhancing Intestinal Function and Nutritional Regulation in Fat Greenling (*Hexagrammos otakii*)

**DOI:** 10.1155/2024/5566739

**Published:** 2024-04-05

**Authors:** Yixin Gu, Yu Zhan, Yafeng Guo, Wenyuan Hua, Xin Qi, Zhizhi Gu, Shengnan Cao, Yan Chen, Zhuang Xue, Wei Wang

**Affiliations:** ^1^Key Laboratory of Applied Biology and Aquaculture of Northern Fishes in Liaoning Province, Dalian Ocean University, Dalian 116023, China; ^2^College of Fisheries and Life Science, Dalian Ocean University, Dalian 116023, China; ^3^Key Laboratory of Biotechnology and Bioresources Utilization, Ministry of Education, Dalian Minzu University, Dalian 116600, China

## Abstract

Cinnamaldehyde is an ideal feed additive with good immune enhancement and anti-inflammatory regulation effects. However, the anti-inflammatory regulation mechanism in fat greenling (*Hexagrammos otakii*, *H. otakii*) remains unclear. The nine targets of cinnamaldehyde were gathered in identified by the Traditional Chinese Medicine Systems Pharmacology database and Uniprot database, and 1,320 intestinal inflammation disease (IIF)-related proteins were screened from DrugBank, Online Mendelian Inheritance in Man (OMIM), Genecards, and Pharmacogenetics and Pharmacogenomics Knowledge Base (PHARMGKB) Databases. According to the Gene Ontology enrichment results and Kyoto Encyclopedia of Genes and Genomes pathway results, cinnamaldehyde may regulated the responses to bacteria, lipopolysaccharide, an inflammatory cytokine, and external stimuli via the nuclear factor kappa-B (NF*κ*B) signaling pathway within on inflammatory network. In addition, the protein–protein interaction analysis assisted in obtaining the closely related inflammatory regulatory proteins, including the C5a anaphylatoxin chemotactic receptor 1 (C5aR1), transcription factor p65 (RELA), prostaglandin G/H synthase 2 (PTGS2), and toll-like receptor 4 (TLR4), which were confirmed as the bottleneck nodes of the network to be more deeply verified via the molecular docking. Moreover, a cinnamaldehyde feeding model was established for evaluating the anti-inflammatory effect of cinnamaldehyde in *vivo*. According to the current findings implied that cinnamaldehyde may play a protective role against IIF *H. otakii* by reducing inflammation through the C5 complement (C5)/C5aR1/interleukin-6 (IL-6) and TLR4/NF*κ*B/PTGS2 pathway. The study focused on investigating the action mechanism of cinnamaldehyde on IIF through combining pharmacology and experimental verification in *vivo*, which provided a fresh perspective on the promoting effect of cinnamaldehyde on IIF in fish.

## 1. Introduction

Fat greenling (*Hexagrammos otakii*, *H. otakii*) refers to a species in Scorpaeniformes, predominantly distributed in China, the Korean Peninsula, and Japan. *H. otakii* refers to an economically essential species because it has rich nutritional content and excellent quality of meat [1]. Consequently, scholars are researching the rapid growth of *H. otakii* to adapt to people's dietary needs. Intensive farming systems have emerged as a promising way to satisfy the growing demand for the species recently. However, fish farming in high-density intensive farming systems poses a considerable risk of causing stress-inducing conditions, which will inflame the intestinal tract of *H. otakii* and make the fish more susceptible to disease, leading to extreme mortality and substantial economic losses [2]. In recent years, there has been a gradual rise in the research on *H. otakii* and intestinal inflammation health. Gu et al. [3] showed that dietary supplementation with 600 mg/kg artemisinin alleviated delayed *Edwardsiella*-induced intestinal immune diseases in *H. otakii* through the superoxide dismutase (Mn), mitochondrial (SOD2)/hypoxia-inducible factor alpha (HIF-1*α*)/nuclear factor kappa-B (NF*κ*B)/vascular endothelial growth factor A (VEGFA) signaling pathway. Fan et al. [4] have demonstrated via macroeconomics that dominant genera in the intestinal, such as *lactococcus*, and boosted with age in Dorado streamers, which in turn improved intestinal health of *H. otakii*. At the same time, many methods have been adopted to control the disease, particularly the employment of antibiotics (Enomycin, flavomycin, halomycin, and bacitracin zinc) [5–7]. Nevertheless, environmental hazards, antibiotic resistance, as well as accumulated residues, primarily restrict the development of intensive aquaculture [8, 9]. Therefore, researchers have paid increasing attention to searching for environmental friendly and safe alternative methods for improving fish intestinal inflammation disease (IIF).

Phytonutrients are an antifree feed additive, with multiple functions and health benefits in the diet [10]. Cinnamaldehyde is a phytonutrient, which is one of the main components of cinnamon (*Cinnamomum zeylanicum*). Cinnamaldehyde has antioxidant [11], anti-inflammatory [12] and anticancer properties [13]. Cinnamaldehyde's bactericidal mechanism has been proven to disrupt bacterial cell membranes, cellular metabolism, and energy production [14]. Moreover, cinnamaldehyde shows anti-inflammatory properties and immune-enhancing effects by inhibiting the secretion of proinflammation cytokines from monocytes and macrophages *in vitro* and *in vivo*, which has been used in traditional medical practice as a supplement to strengthen the immune system and fight various infectious diseases [12, 15]. Recently, people have paid more and more attention to the positive effect of cinnamaldehyde additives on animal health, and researchers have reported such positive effect in aqua feed in various fish species including Nile tilapia (*Oreochromis niloticus*) [16], Channel catfish (*Ictalurus punctatus*) [17], *Channa striatus* [18], Tongue sole (*Cynoglossus semilaevis*) [19], juveniles *Cyprinus carpio* [20], and Zebrafish (*Danio rerio*) [21]. Also, according to the study of Zhou et al. [22], supplementing 144 mg/kg of cinnamaldehyde in the diet of grass carp (*Ctenopharyngodon idella*) improved IIF by activating the NF*κ*B signaling pathway, thereby increasing digestive enzyme activities. Chen et al. [23] pointed out that cinnamaldehyde can boost intestinal health and digestion levels by reducing IIF and increasing the abundance of beneficial bacteria. Cinnamaldehyde has the primary benefit of elevating fish performance by relieving inflammation and enhancing intestinal function and nutrient digestibility [24]. Although there is evidence of a beneficial impact of cinnamaldehyde, the precise mechanism of action in fish is not well understood.

Systems pharmacology is an emerging field of integrated analysis through pharmacology, pharmacodynamics, and drug target networks, the systematic of which are consistent with the trellis coded (TCM) modulation principle. Systemic pharmacology is major applied in medical and pharmaceutical research, investigating the mechanisms of action of drugs in the treating human diseases [25]. However, systemic pharmacology has been comparatively little studied in aquaculture [3]. According to our previous experiments, cinnamaldehyde at 600 mg/kg increased intestinal digestive enzyme activities, apparent digestibility coefficient, and the weight gain rate in juvenile *H. otakii* [26]. Here, network pharmacology is proposed for investigating the molecular mechanisms regarding cinnamaldehyde by screening active compounds, as well as predicting therapeutic targets. In addition, systematic pharmacological studies and experimental validation provide the necessary data for exploring the pharmacological effects and potential mechanisms of cinnamaldehyde for ameliorating IIF in *H. otakii*. The results of this study will offer useful fundamental information and a point of reference for the creation of cinnamaldehyde as a precise nutritional need for fish farming.

## 2. Materials and Methods

### 2.1. Putative Target Protein (PTP) and IIF Related Proteins Screening

We searched the bioactive ingredients of cinnamaldehyde through the Tradition Chinese Medicine Systems Pharmacology Database and Analysis Platform (TCMSP, https://tcmspw.com/tcmsp.php) by a search keyword of “Cinnamaldehyde” in English, and obtained the corresponding targets of cinnamaldehyde by molecular identity document (ID). The target proteins' gene names came from the Uniprot database (https://www.uniprot.org/). We set the “Organisms” to “Zebrafish (*Danio rerio*).”

The search entry for the target disease was “intestinal inflammation.” The DrugBank (https://go.drugbank.com), Online Mendelian Inheritance in Man (OMIM) (https://omim.org), Genecards (https://www.genecards.org), as well as Pharmacogenetics and Pharmacogenomics Knowledge Base (PHARMGKB) databases (https://www.pharmgkb.org) served for acquiring relevant targets. A relevance score ≥10 was taken as the screening criterion to retrieve active targets from the Genecards database.

### 2.2. Gene Ontology (GO) and Kyoto Encyclopedia of Genes and Genomes (KEGG) Pathway Enrichment and the Constructions of Network

The gene ontology (GO) and Kyoto Encyclopedia of genes and genomes (KEGG) enrichment analysis using the R package based on the [27]. Sum up, the core target proteins were imported into R packages. The study conducted GO and KEGG enrichment analysis under the assistance of clusterProfiler packages and org.Hs.eg.db packages provided by Bioconductor. The threshold of the *P* value was 0.05, and the correction method was a false discovery rate (FRD). The enrichment results were arranged in ascending order of *P* value, and the top terms were selected for plotting.

Our team members entered the targets of cinnamaldehyde and the disease targets of IIF into the VENNY 2.1.0 platform (https://bioinfogp.cnb.csic.es/tools/venny) to acquire the intersecting targets. A Wayne diagram was then drawn. We imported the obtained intersecting targets into the STRING 11.0 platform (https://cn.string-db.org), and set the species as “Zebrafish” and the “medium confidence” as 0.400. That was followed by the construction of the protein–protein interaction (PPI) network. Then, the PPI network diagram file was downloaded (PDB format). With the purpose of constructing and analyzing the “component-target” network diagram of cinnamaldehyde, the downloaded file was imported into Cytoscape 3.8.2 software, and the relationship between targets were calculated by clicking “tools and analyze network.” The nodes represent drugs, genes, and active ingredients, and the edges represent the corresponding relationships [28].

### 2.3. Molecular Docking

The core target proteins of juvenile *H*. *otakii* underwent homology modeling with the assistance of SWISS-MODEL sever (https://swissmodel. expasy.org) in alignment mode. The server constructed a model considering the alignment between the target and the known templates in the protein data bank (PDB; https://www.rcsb.org/) [29]. However, its method was invalid due to the distinct association between the sequence and the structural database available. Accordingly, the sequence presented poor amino acid homology. Therefore, another software Alphafold 2(https://colab.research.google.com/github/sokrypton/ColabFold/blob/main/Alphafol2.ipynb#) was adopted for calculating the high-precision construction based on the target protein sequence [30].

ChemDraw 3D software served for converting the ligands ethyl cinnamaldehyde (Pubchem) into PDB coordinate flies [31]. The ligand followed Lipinski's “rule of five” which targets drug-like properties [32]. DeepSite (https://www.playmolecule.com/deepsite/) predicted each protein's binding sites. The search grid of the Auto Dock Vina 1.5.7 software assisted in simulating the ligand entering proteins' active site, thereby calculating the ligand–receptor complex's binding energy (The information of molecular docking coordinates and BOX: C5aR1: center *x* = 5.628, center *y* = −9.825, center *z* = −8.697; size *x* = 69.65, size *y* = 69.65, size *z* = 69.65; p65: center *x* = 11.181, center *y* = −0.757, center *z* = −7.066, size *x* = 126.0, size *y* = 126.0, size *z* = 126.0; TLR4: center *x* = 11.181, center y = −0.757, center *z* = −7.066, size *x* = 59.85, size *y* = 59.85, size *z* = 59.85;and PTGS2: center *x* = 11.181, center *y* = −0.757, center *z* = −7.066, size *x* = 97.65, size *y* = 97.65, size *z* = 97.65). The computer docking was taken into account for predicting nine docking positions specific to each ligand–protein complex. For the preferred compound binding direction, the binding affinity score was more negative, which shall be further studied. We chose the value with the largest absolute value of the binding energy as the optimal docking position for the ligand and receptor [33]. PyMOL and LigPlot 2.2.5 served for the visualization of the three-dimensional (3D) and two-dimensional (2D) docking structures, respectively [34].

### 2.4. Experimental Diets and Design

Table 1 lists experimental diet formulation and proximate composition. Fish meal and chicken gut meal were the primary sources of protein, and fish oil was the primary source of fat. Experimenters formulated six experimental diets with cinnamaldehyde at 0, 200, 400, 600, 800, and 1,000 mg/kg diet, namely (CNE0, CNE200, CNE400, CNE600, CNE800, and CNE1000), the dose of cinnamaldehyde were based on [26]. The experimental feed preparation was based on the method described in detail in [22]. In other words, the milled feed was placed in a Hobart type mixer (F-26, South China University of Technology Machine Works) to produce cold extruded pellets and the pellet strands were cut into uniform sized pellets with diameters of 2 and 4 mm. After about 24 hr of oven drying at 43°C, polythene bags-sealed pellet feeds were stored at −20°C.

### 2.5. Experiment Feeding Management and Sample Collection

*H. otakii* was provided by the key laboratory of applied biology and aquaculture of fish (Dalian, China). The experimenters assigned 270 healthy juvenile fishes (6.21 ± 0.19 g) to 18 (30 cm × 75 cm) cages in the circulating groove (per disinfection) randomly, and assigned each diet to three replicate groups of fish in a random manner. The experiment fish underwent one week of feeding acclimation before the experiment. In the feeding trial that lasted 8 weeks, fish were fed at 9:00 and 16:00 in a natural photoperiod. Experimental conditions were water temperature: 10 ± 2°C, salinity: 26–30, potential of hydrogen (pH): 7.8 ± 0.4, dissolved oxygen: 6.6 ± 0.7 mg/L, and ammonia nitrogen content <0.1 mg/L. After the feeding trial that lasted 8-week, eight healthy fish from each group (15 fish per tank) received the anesthetization of 100 mg/L Methane-Sulfo-nate-222 (MS-222, Sigma) to measured the growth performance. The obtained intestine was placed in liquid nitrogen after quick freezing (−80°C) for gene expression assays (six fish per bucket, *n* = 3). On the other hand, the fresh liver samples were obtained from two fish from each bucket and preserved in 4% cell fixative for preparation of paraffin sections. Percent weight gain (PWG, %) = 100 × (final weight−initial weight)/initial weight; Feeding rate (FR, %/d) = feed intake in dry matter/100 × ((initial body weight + final body weight)/2)/feeding trial days.

### 2.6. Dietary Proximate Composition Analysis

The nutrient content measurements of the *H. otakii* diets were based on the [35]. In general, moisture was estimated by drying to constant weight in a constant temperature oven at 105°C, and ash was calculated in a muffle furnace at 550°C for 5 hr. At first, crude protein (*N* × 6.25) was first digested with concentrated sulfuric acid and subsequently determined using the Kjeldahl method. Through petroleum ether extraction, the crude lipid was analyzed (Soxhlet extraction).

### 2.7. Histopathology

The intestine histology is based on [36]. In brief, the intestines immersed in Bouin's solution cryopreservation tubes were rinsed with 75% alcohol, placed in embedding cassettes, and dehydrated in a fully automated tissue dehydrator (Joy's Instruments and Equipment Co. Shanghai, China). The dehydrator tissues were embedded in already melted paraffin and sliced (5 *μ*m) using a slicer (Joy's Instruments and Equipment Co. Shanghai, China), attached to slides, and cauterized for 12 hr at 50°C. Staining was performed in a tissue stainer, followed by sealing the sections with neutral resin and drying at room temperature. The microscope (Nikon YS100, Japan) was utilized for observing the intestinal morphology and structure of the six groups.

### 2.8. Quantitative Real-Time PCR (qRT-PCR) Analysis

The TRIzol method assisted in extracting RNA from the intestine of juvenile *H. otakii* [37]. Ultramicro photometer (Biochrom Technologies, Switzerland) served for assessing the total RNA quality and quantity. All samples presented a 260/280 nm absorbance ratio in the 1.85–2.00 range. The reverse transcription kit served for synthesizing cDNA using total RNA as a template and the synthesized cDNA was preserved at −20°C (Baisai Biotechnology, Shanghai, China).  Table 2 lists the primer sequences. Experimenters took *β*-actin as a housekeeping gene, and primers of *β*-actin and toll-like receptor 4 (TLR4) as basis [38]. The fluorescence quantitative PCR reaction system involved 20 *μ*L: 0.6 *μ*L upstream primer, 0.6 *μ*L downstream primer, 10 *μ*L 2 × Talent qPCR PreMix, 1 *μ*L cDNA, and 7.8 *μ*L RNase-Free ddH_2_O. A quantitative thermal cycler assisted in the qRT-PCR analysis (Roche, Light cycler 96, Basel, Switzerland). The cycling conditions for qRT-PCR: 3 min at 95°C, 40 cycles for 15 s annealing at 60°C, as well as 5 s denaturation at 95°C. Melting curve analysis was conducted in a temperature increment range of 55−95°C. The final product underwent agarose gel electrophoresis, confirming that there were single amplicons. The six dilutions (in triplicate) were used for generating the standard curves. The 2^−*ΔΔ*CT^ method analyzed the expression data [39].

### 2.9. Statistical Analysis

Under the assistance of the software SPSS 19.0, the one-way ANOVA served for experiment data analysis. Data were in the form of mean ± standard error of the mean (SEM). Kolmogorov–Smirnov test and Levene's test, respectively helped to test the distribution normality and the variance homogeneity of raw data. Mathematical transformations would be employed in the case that at least one assumption failed to be verified. The comparisons were conducted between each treatment group and the control group,  ^*∗*^*P* < 0.05;  ^*∗∗*^*P* < 0.01;  ^*∗∗∗*^*P* < 0.001.

## 3. Results

### 3.1. Integrated Network Model Construction and Analysis

**Figure 1:**
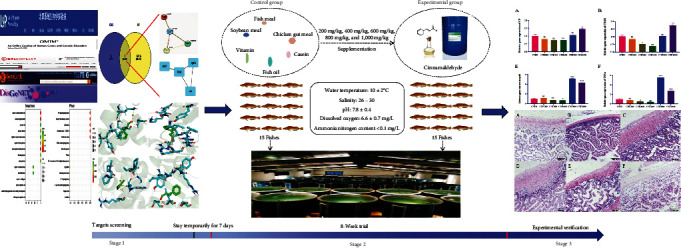
The flowchart of the experimental culture trial and exercise workflow diagram of in the ameliorate of intestinal inflammation disease (IIF). Stage 1—screening and selection of targets for cinnamaldehyde; Stage 2— adding different concentrations of cinnamaldehyde to feed the *H. otakii*; Stage 3—experimental validation against selected targets. All tests were performed in laboratory conditions. The relevant targets of cinnamaldehyde and intestinal inflammation were first searched through drug and disease-related databases, followed by Wayne plots, enrichment analysis, and PPI network analysis to establish the targets at the intersection of cinnamaldehyde and intestinal inflammation as well as their interrelationships. Subsequently, we conducted *in vivo* experiments by supplementation of cinnamaldehyde to the diets of *H. otakii*, and finally validated the predicted targets by observing the histological morphology and structure and quantifying the inflammatory genes.

**Figure 2:**
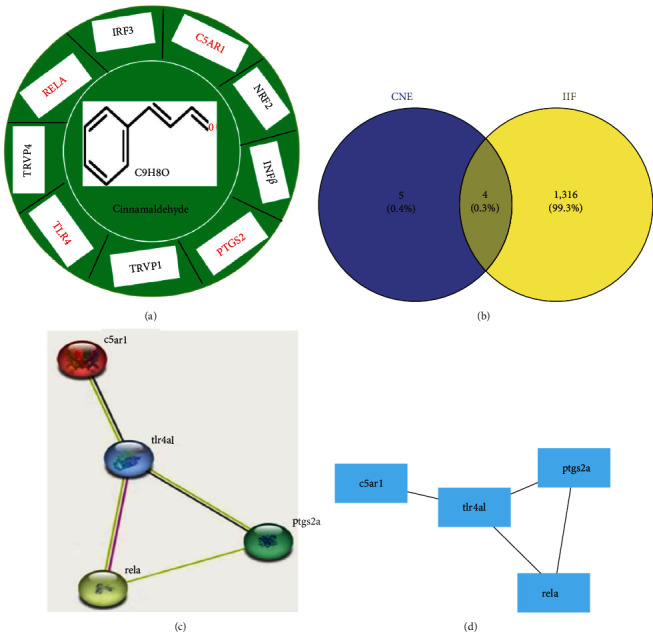
Comparison and intestinal inflammation disease (IIF) and cinnamaldehyde targets: (a) cinnamaldehyde-target network construction, and cinnamaldehyde putative target protein (PTP) classification; (b) Venny 2.1.0 diagram of IIF and cinnamaldehyde targets; (c) the “Cinnamaldehyde—Target—IIF” network diagram. STRING 11.0 analyzed the protein–protein interaction (PPI) network of IIF and cinnamaldehyde targets. Network nodes and edges respectively denote proteins and protein relationship. Known interactions: light blue and pink edges represent curated database and experimentally determined, respectively; yellow and black edges represent text-mining and co-expression, separately; and (d) Cytoscape 3.8.2 verified PPI network.

Figure 1 displayed the potential action mechanisms in defining the effect of cinnamaldehyde on IIF by system pharmacology and experimental verification together with the details behind each step. We retrieved 12 gene targets from TCMSP database species, and obtained nine bioactive targets by screening the gene targets of “Zebrafish” in Uniprot software (Figure 2(a)).

We respectively obtained 108, 29, 668, and 682 targets from Drugbank, OMIM, Genecards, and PHARMGKB databases, and obtained 167 action targets after duplicate removal. The obtained targets corresponded to nine targets of cinnamaldehyde (*Supplementary 1*). The VENNY 2.1.0 platform mapped the obtained four intersecting targets at last (Figure 2(b)).

Then, we imported the four intersecting targets into the STRING 11.0 database, and obtained the relationship score over 0.98. Figures 2(c) and 2(d) displayed the PPI network. The network has four proteins with two relationships. The four key targets are C5a complement receptor 1 (C5aR1), transcription factor p65 (RELA), prostaglandin G/H synthase 2 (PTGS2), and TLR4.

### 3.2. Cinnamaldehyde Molecular Action Mechanism

**Figure 3:**
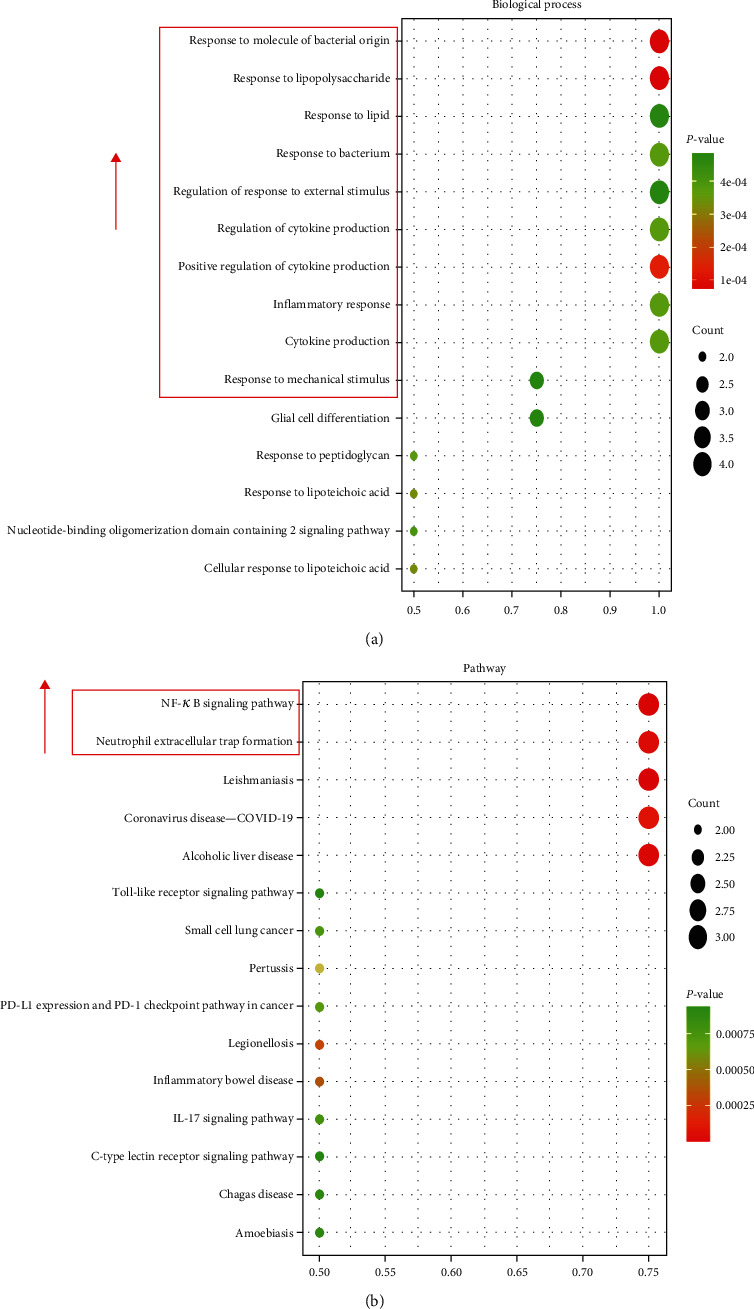
Enrichment analysis of pathways and processes of the core targets of cinnamaldehyde to alleviate intestinal inflammation disease (IIF) using the R package: (a) Gene ontology (GO)—biological process (BP) functional enrichment analysis and (b) Kyoto Encyclopedia of genes and genomes (KEGG) signaling pathway enrichment analysis. The items were sorted from high to low according to the *P* value of 0.05, and the top 15 bp items were filtered out and plotted as bubble plots.

The four intersecting targets received GO enrichment analysis after being imported into the R packages. The *P* value was taken into account to select the GO enrichment rank entries, thereby drawing a 2D bubble chart (Figure 3(a)). There were 833 enrichment results, and 780 (93.64%) key targets primarily concentrated upon biological processes (BP). In the GO analysis, the top 15 BP terms were the response to bacterial origin molecule, to lipopolysaccharide, to lipid, to the bacterium, to mechanical stimulus and external stimulus, the cytokine production regulation, positive cytokine production regulation, inflammatory response, and cytokine production.

Four core targets between cinnamaldehyde and IIF were imported into the R package to receive the KEGG pathway enrichment analysis, with 62 projects generated. We drew the 2D bubble chart by the top 15 projects with P values (Figure 3(b)). Hence, the NF*κ*B signaling pathway and neutrophil extracellular trap formation were the top two. Each pathway has various targets (Table 3).

### 3.3. Molecular Docking

**Figure 4:**
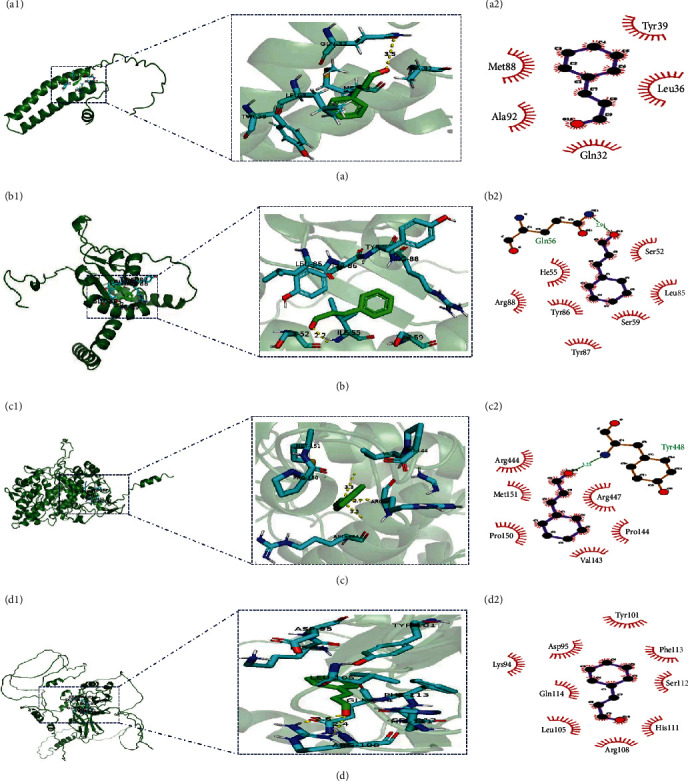
The three-dimensional (3D) and two-dimensional (2D) schematic diagrams of molecular docking models, active sites, and binding distances of the core target proteins of cinnamaldehyde molecules in *H. otakii*, as well as the name of amino acid residues and hydrogen bonding positions. The ray tracing of: (a) Cinnamaldehyde with C5a complement receptor 1 (C5aR1), (b) cinnamaldehyde with toll-like receptor 4 (TLR4), (c) cinnamaldehyde with prostaglandin G/H synthase 2 (PTGS2), and (d) cinnamaldehyde with transcription factor p65 (p65), respectively.

The four targets of C5aR1, TLR4, RELA, and PTGS2 underwent molecular docking with cinnamaldehyde (Figure 4 and Table 4). The binding energy of the C5aR1 and cinnamaldehyde is −4.8 Kcal mol^−1^ with one hydrogen bond and five amino acid residues in the hydrophobic interactions. The TLR4 interacted with cinnamaldehyde through the hydrophobic interactions involving one hydrogen bond and seven amino acid residues, at a binding energy of −4.6 Kcal mol^−1^. The RELA exhibited an intense affinity with cinnamaldehyde through the hydrophobic interactions involving two hydrogen bonds and nine amino acid residues, and the binding energy was −4.7 Kcal mol^−1^. The PTGS2 interacted with cinnamaldehyde through three hydrogen bonds and six hydrophobic interactions, and the binding energy was −5.2 Kcal mol^−1^.

### 3.4. Cinnamaldehyde Boosts Intestinal Structure of *H. otakii*

**Figure 5:**
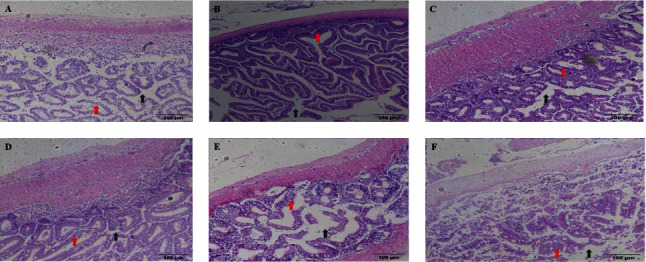
Dietary cinnamaldehyde boosted in intestinal healthy. The intestinal histopathology for hematoxylin–eosin (H&E) staining: (A) CNE0 group; (B) CNE200 group; (C) CNE400 group; (D) CNE600 group; (E) CNE800 group; and (F) CNE1000 group. The arrows in the figure indicate damage to the intestinal villi, intestinal wall sites. Red arrows: intestinal villi; black arrows: intervillous space.

Figure 5 presents the morphological structure of the intestinal tract. Compared with the CNE0 group, the CNE200, CNE400, and CNE600 groups showed a decreased in intestinal inflammatory cell infiltration, intestinal mucosal morphology tended to be intact, and the intestinal villi were increased and densely and compactly arranged. In the CNE800 and CNE1000 groups, the intestinal inflammatory cell infiltration was increased, and the intestinal villi became fewer, with an increased in the intervillous space, and in the CNE1000 group, some of the intestinal villi had been detached and incomplete, and the arrangement of the villi was disorganized.

### 3.5. Inflammation

**Figure 6:**
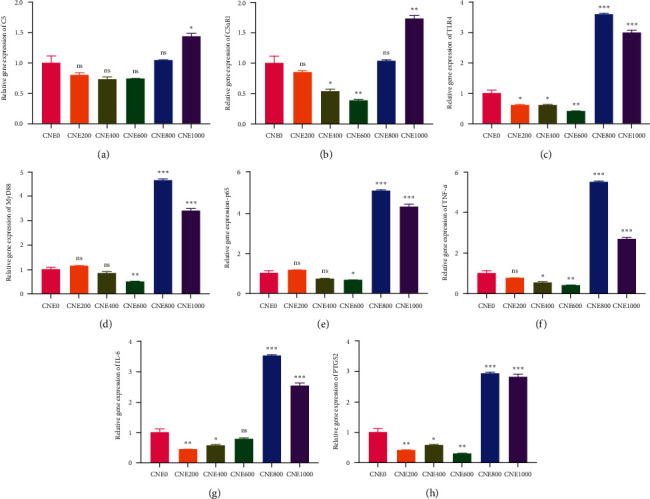
Impacts of dietary cinnamaldehyde on inflammation-related gene expression in *H. otakii* intestine: (a) *C5*, C5 complement; (b) *C5aR1*, C5a complement receptor 1; (c) *TLR4*, toll-like receptor 4; (d) *MyD88*, myeloid differentiation primary response gene 88; (e) *p65*, transcription factor p65; (f) *TNF-α*, tumor necrosis factor alpha; (g) *IL-6*, interleukin 6; and (h) *PTGS2*, prostaglandin G/H synthase 2. The identical indexes with  ^*∗*^ and  ^*∗∗*^ had obviously different mean values (*P* < 0.05, *P* < 0.01), and the “not significantly (ns)” denotes that there were no remarkable differences between groups.

Figure 6 shows the relative gene expression of the intestinal tract. The mRNA levels of *TLR4*, *interleukin-6* (*IL-6*), and *PTGS2* in the CNE200 group presented an obvious decreased of about 1.66, 2.30, and 2.35 folds greater in to the control group (*P* < 0.05). The mRNA levels of *C5aR1*, *TLR4*, *tumor necrosis factor alpha* (*TNF-α*), *IL-6*, and *PTGS2* were markedly decreased by approximately, 1.87, 1.63, 1.84, 1.77, and 1.66 folds greater in CNE400 group (*P* < 0.05). The mRNA expressions of *C5aR1*, *TLR4*, *myeloid differentiation primary response gene 88* (*MyD88*), *transcription factor p65* (*p65*), *TNF-α*, and *PTGS2* were significantly decreased by approximately 2.59, 2.30, 2.00, 1.51, 2.46, and 3.16 folds greater in CNE600 group relative to CNE0 group (*P* < 0.05). The mRNA levels of *TLR4*, *MyD88*, *p65*, *TNF-α*, *IL-6*, and *PTGS2* were notably increased by approximately 3.57, 4.62, 5.06, 5.44, 3.49, and 2.91 folds greater in CNE800 compared with the control, and their levels were markedly increased by approximately 1.43, 1.71, 2.98, 3.37, 4.24, 2.65, 2.52, and 2.80 folds greater in CNE1000 group in relative to CNE0 group (*P* < 0.05). The mRNA expressions of *C5 complement* (*C5*) and *IL-6* were increased by approximately 1.36 and 1.26 folds greater in CNE600 compared with the control (*Supplementary 2* and *3*). In addition, the PWG and FR were significantly increased with dietary cinnamaldehyde supplementation (*P* < 0.05; *Supplementary 4*).

## 4. Discussion

For the purpose of investigating the action mechanism of cinnamaldehyde action on IIF in *H. otakii*, the study adopted network pharmacology, and molecular docking together with experimental validation. Moreover, network pharmacology can thoroughly investigate the constituents and targets of drugs is crucial for analyzing their effects [40]. In this study, nine drug targets were obtained by searching the TCMSP and Uniprot databases. Cinnamaldehyde, the primary active component in cinnamon, was identified to have four common targets with IIF. These targets, namely C5aR1, RELA, TLR4, and PTGS2, were screened and used in constructing the PPI network. In addition, the terms of BP identified by hub gene GO enrichment analysis indicated the obvious relation between the core targets and the response to the bacterium, lipopolysaccharide (LPS), an inflammatory cytokine, as well as external stimulus, which are consistent with the pathological process of IIF [41–43]. Expect for that, the KEGG pathway further demonstrated that four core targets were mainly enriched in the formation of neutrophil extracellular trap and NF*κ*B signaling pathway. Crucially, the favorable outcomes of molecular docking served as additional confirmation that cinnamaldehyde exerts a robust effect in mitigating IIF. This established a solid foundation for subsequent fish experiments. Molecular docking verified the ability of cinnamaldehyde to bind to these four target proteins well. The results showed that the core target protein PTGS2 has the strongest binding affinity for cinnamaldehyde. The hydrogen bonds formed by these four core target proteins with cinnamaldehyde were more stable, allowing the stable binding of the cinnamaldehyde ligand to relevant proteins' active sites. On the other hand, small molecular ligands underwent hydrophobic interactions with protein residues, which can enhance compound stability in the active pocket [44]. However, molecular docking can only be used to predict the binding state between drug molecules and certain receptors, and cannot predict the metabolism and efficacy of drug molecules in organisms. Therefore, molecular docking can only play an auxiliary and guiding role, and experiments are required to demonstrate the results of molecular docking.

The intestine can crucially affect the nutrient digestion and disease defense in fish and takes charge of approximately 70% of fish immune function. In accordance with Dawood et al. [45], the digestive and absorptive capacity of the fish intestine is the primary factor directly influencing its growth performance. In addition, numerous nutritional studies have pointed out that a quality dietary formulation strategy can enhance the efficient digestion of nutrients by the intestine, thus increasing the rate of weight gain and specific growth rate in fish [46–48]. The intestine of fish primarily takes charge of nutrient digestion, which is also an essential barrier to preventing antigens and pathogens from entering the fish [49]. Especially in intensive aquaculture systems, IIF has become one of the factors limiting fish digestion and growth. The IIF directly reflects the degree of damage to the intestinal mucosal barrier by pathogenic bacteria [50]. Inflammation is accompanied by an upregulation of proinflammatory cytokines and a decreased of anti-inflammatory cytokines in fish [51]. The imbalanced activity of cytokines disrupts the relative homeostasis of the microbial community in the gut, leading to disruption and impairment of the bacterial flora, which diminished digestive enzyme activity and hinders digestive capacity [52]. In the CNE200, CNE400, and CNE600 groups, the intestinal inflammatory cells were reduced, the villi were increased and intact, and the villi were relatively dense and compact, which improved the intestinal health. This suggested that cinnamaldehyde can improve the intestinal structure to alleviate IIF in *H. otakii*.

Studies have shown that IIF can activated the complement system disrupting the gut microbiome [53]. The complement system is an important self-defense immune system, and its activation contributes to the aggregation of macrophages and phagocytosis of foreign bacteria [54]. Three types of pathways can activate the complement system classical, alternative, and lectin [55]. The activation of the classical pathway relies on the antigen–antibody complex. Alternative pathways are initiated directly by surface molecules of bacteria and fungi. The lectin pathway involves the binding of mannose-binding lectin proteins to the carbohydrate structure of bacteria, thereby activating serine proteases, and binding to complement proteins [56, 57]. All three pathways result in the activation of C5 and the formation of a membrane attack complex, resulting in the cleavage of the C5 to C5a complement (C5a) [58]. Among complement-activated products, C5a, among complement-activated products, stands out as one of the most potent inflammatory peptides with a diverse array of functions. C5a fragments are powerful proinflammatory factors produced during complement activation and exert their biological functions through interaction with the specific receptor C5aR1 [59]. Numerous studies have indicated that blocking the C5a/C5aR1 signaling pathway can prevented the occurrence of IIF and enhanced survival, demonstrating the importance of C5a/C5aR1 in regulating the pathological process of IIF [60–62]. Then, the activation of the C5a/C5aR1 signaling pathway promotes neutrophil and macrophage infiltration in perivascular adipose tissue, and induced pro-inflammatory M1 macrophage polarization and inflammatory cytokine expression such as IL-6 [63, 64]. IL-6 is a major proinflammatory cytokine [65]. According to Jain et al. [66], which findings illustrated that C5a directly stimulated IL-6 generation in colonic epithelial cells, and suppression of C5a in infected wild-type mice results in defective epithelial IL-6 output and exacerbates inflammation. Additionally, TLR4, a member of the toll-like receptor family, is expressed on the cell surface of macrophages and neutrophils [67]. TLR4 is likely the key mediator of lipopolysaccharide (LPS) signaling in IIF. During intestinal inflammation, LPS interacts with TLR4 in neutrophils and macrophages [68–70]. After recognizing, pathogen-related molecular patterns (LPS), TLR4 is connected to initiation signals through its cytoplasmic tail toll/IL-1R (TIR) structure. This domain is capable of recruiting the convertor protein myeloid MyD88, which activates the NF*κ*B signaling pathway as well as promotes downstream proinflammatory cytokines (TNF-*α* and PTGS2) to be released [71–73]. The p65 is a combined form of NF*κ*B protein [64], which crucially impacts IIF pathogenesis [74, 75]. TNF-*α* is the earliest endogenous mediator of inflammatory response. Studies have shown that endotoxemia induces intestinal mucosal injury as well as chronic inflammatory bowel disease by upregulating TNF-*α* expression [65]. PTGS2 is one of the key factors in the cellular response to inflammation. Silencing of PTGS2 expression has been reported to attenuate LPS-induced inflammatory responses by negatively regulating the NF*κ*B signaling pathway [76]. Here, the supplementation of CNE at 600 mg/kg lowered the mRNA levels of *C5*, *C5aR1*, *TLR4*, *MyD88*, *p65*, *TNF-α*, *IL-6*, and *PTGS2* in *H. otakii* intestinal tract, which is the similar to the finding of [22]. Hence, cinnamaldehyde could improve IIF in *H. otakii*. Nevertheless, *in vivo* experiments in fish to validate the targets predicted by network pharmacology are not comprehensive enough. Therefore, *in vitro* experiments and further validation of target function will be the focus of future research by nutritionists.

## 5. Conclusion

**Figure 7:**
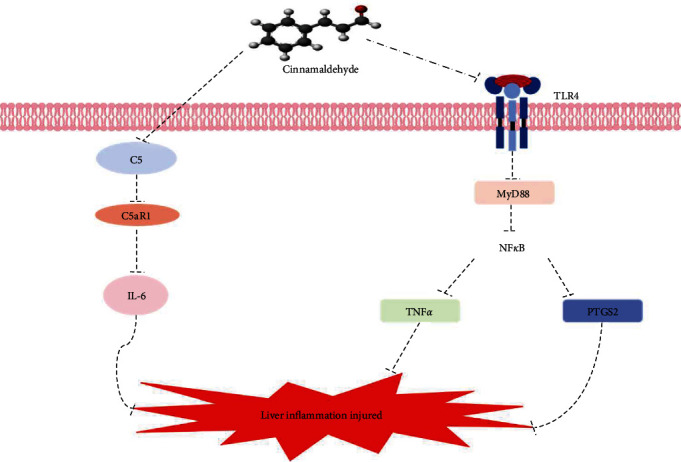
Inflammation system interaction of the Kyoto Eencyclopedia of genes and genomes (KEGG) enrichment pathway terms and putative target protein (PTP) in the intestinal inflammation disease (IIF) of *H. otakii* and cinnamaldehyde. Cinnamaldehyde may restrict C5/C5aR1/IL-6 and TLR4/NF*κ*B/PTGS2 pathway by limiting the C5, C5aR1, and IL-6, and also inhibits TLR4, MyD88, NF*κ*B, and primary inflammatory cytokines (TNF-a and PTGS2), that can help to relive the intestinal inflammation and maintaining intestinal function.

To conclude, this current work indicates the possible action mechanism of cinnamaldehyde in IIF via a network pharmacological approach, that assists in evaluating the protective mechanism of cinnamaldehyde on *H. otakii* intestine from a theoretical perspective (C5/C5aR1/IL-6 and /TLR4/NF*κ*B/PTGS2; Figure 7). The supplementation of cinnamaldehyde at 600 mg/kg in diets significantly improved the anti-inflammatory ability and promoted digestion and growth (Data not present). In summary, dietary cinnamaldehyde is an effective additive to promote the intestinal health of *H. otakii* as well as improve IIF. This study reveals for the first time the underlying mechanism of action of cinnamaldehyde in improving the IIF of *H. otakii* by means of systemic pharmacology, molecular docking, and experimental validation, which is significant for the healthy culture of *H. otakii* streamers and the promotion of new feed additives in the future.

## Figures and Tables

**Table 1:** The formulation the diets in *H. otakii* (% dry matter).

Ingredients	Contents (%)
Fish meal^a^	15.48
Chicken gut meal^b^	24.52
Soybean meal^c^	30
Casein^d^	14.8
Fish oil^e^	5
Flours^f^	3
Corn starch^g^	4
Cr_2_O_3_^h^	0.2
Vitamin premix^i^	1
Mineral premix^j^	1
Sodium alginate^k^	1
Total	100
Proximate composition (%)	—
Moisture	9.92
Protein	50.55
Lipid	10.41
Ash	8.06

*Note*. ^a^Fish meal has a crude protein of 58% and a crude lipid of 7.2%, which selected with Meiweiyuan Biotechnology Co., Qingdao, Shandong, China. ^b^Chicken gut meal has a crude protein of 65.47% and a crude lipid of 14.8%, which selected with Yufeng Feed Co., Anshan, Liaoning, China.^c^Soybean meal has a crude protein of 42.5% and a crude lipid of 2.1%, which selected with Meiweiyuan Biotechnology Co., Qingdao, Shandong, China. ^d^Casein has a crude protein of 86.2% and a crude lipid of 1.5%, which selected with Meiweiyuan Biotechnology Co., Qingdao, Shandong, China. ^e^Fish oil selected with Meiweiyuan Biotechnology Co., Qingdao, Shandong, China. ^f^Flours has a crude protein of 6.2% and a crude lipid of 0.9%, which selected with Meiweiyuan Biotechnology Co., Qingdao, Shandong, China. ^g^Corn starch has a crude protein of 0.3% and a crude lipid of 0.1%, which selected with Meiweiyuan Biotechnology Co., Qingdao, Shandong, China. ^h^Cr_2_O_3_ selected with McLin Biotech Co., Shanghai, China. ^i^Vitamin premix per kilogram contains vitamin of 7,000 IU, vitamin E of 50 mg, vitamin D_3_ of 2,000 IU, vitamin K_3_ of 10 mg, vitamin B_1_ of 20 mg, vitamin B_2_ of 20 mg, vitamin B_6_ of 30 mg, vitamin B_12_ of 0.1 mg, nicotinic acid of 80 mg, vitamin C of 100 mg, Ca pantothenate of 50 mg, folic acid of 6 mg, and inositol of 80 mg. ^j^Mineral premix per kilogram contains MgSO_4_·7H_2_O of 5,782 mg, FeSO_4_·7H_2_O of 1,000 mg, NaCl of 3,000 mg, ZnSO_4_·7H_2_O of 150 mg, MnSO_4_·4H_2_O of 50.3 mg, CuSO_4_·5H_2_O of 15 mg, CoCl_2_·6H_2_O of 1.2 mg, and KI of 1.5 mg. ^k^Sodium alginate selected with Meiweiyuan Biotechnology Co., Qingdao, Shandong, China.

**Table 2:** Sequences of primers used in qRT-PCR.

Gene	Primer sequence (5′–3′)
*C5 - F*	GCGACTCCTCTGGTTGTATAG
*C5 - R*	GAACAGGAAGTGAGCGTATGT
*C5aR1 - F*	GAACACGAGGCCGTAGAAG
*C5aR1 - R*	GGAGACTTCAATTCCACCAATTAC
*TLR4 - F*	GGAATGTTGCTCAGTTGTCTCT
*TLR4 - R*	CAGGCGAGTCAGATACTTCAGA
*P65 - F*	GACTGCAAACACGGCTACTA
*P65 - R*	GGCCTCATTCACATCCTTCTT
*PTGS2 - F*	CATTGAAGGTCGGAGGACTATC
*PTGS2 - R*	CGTCAGCAACATCTCCTTCT
*MyD88 - F*	TAGTCGCATATGGTGAGGAAAC
*MyD88 - R*	GTCCCGGAGCTGAAAGTAAA
*TNF-α - F*	CTTCTACCAGTACGCACATCC
*TNF-α - R*	AACACTCAGACAGCCATACAC
*IL-6 - F*	GTCTGTATCTGGCCGTGATATG
*IL-6 - R*	ATGACCGTTACCTGGAGTTTG
*β - Actin*	CTGGTCTGGATTGGCTGTGA
*β - Actin*	GGAAGGAAGGCTGGAAGAGG

*Abbreviations. C5*, C5 complement; *C5aR1*, C5a complement receptor 1; *TLR4*, toll-like receptor 4; *p65*, transcription factor p65; *PTGS2*, prostaglandin G/H synthase 2; *MyD88*, myeloid differentiation primary response gene 88; *TNF-α*, tumor necrosis factor alpha; *IL-6*, interleukin 6.

**Table 3:** KEGG analysis of the 15 pathways.

ID	Pathway	Gene
Hsa04064	NF*κ*B signal pathway	RELA, PTGS2, TLR4
Hsa04613	Neutrophil extracellular trap formation	TLR4, C5aR1, RELA
Hsa05140	Leishmaniasis	PTGS2, TLR4, RELA
Hsa05171	Coronvavirus disease—COVID-19	C5aR1, TRL4, RELA
Hsa04936	Alcoholic liver disease	TLR4, RELA, C5aR1
Hsa04620	Toll-like receptor signaling pathway	TLR4, RELA
Hsa05222	Small cell lung cancer	PTGS2, RELA
Hsa05133	Pertussis	TLR4, RELA
Hsa05235	PD-L1 expression and PD-1 checkpoint pathway in cancer	TLR4, RELA
Hsa05134	Legionellosis	TLR4, RELA
Hsa05321	Inflammatory bowel disease	TLR4, RELA
Hsa04657	IL-17 signaling pathway	PTGS2, RELA
Hsa04625	C-type lectin receptor signaling pathway	PTGS2/RELA
Hsa05146	Amebiasis	TLR4/RELA

**Table 4:** The results of molecular docking.

Gene	Binding energy (KJ/mol)	Hydrogen	Amino acid residues
C5aR1	−4.8	1	5
RELA	−4.7	2	9
PSTG2	−5.2	3	6
TLR4	−4.6	1	7

## Data Availability

The data that support the findings of this study are available from the corresponding author upon reasonable request.

## Supplementary Materials

Supplementary 1 The information of target genes in manuscript.

Supplementary 2 The gene sequences of fat greenling used in the manuscript.

Supplementary 3 The protein sequences of fat greenling used in the manuscript.

Supplementary 4 The percent weight gain and feeding rate of *H.otakii* for feeding 8-week.
