# Supplementary material for: Potential Targets and Signaling Mechanisms of Cinnamaldehyde Enhancing Intestinal Function and Nutritional Regulation in Fat Greenling (Hexagrammos otakii)
Source: Aquac Nutr. 2024 Apr 5;2024:5566739. doi: 10.1155/2024/5566739 (PMC11074912; doi:10.1155/2024/5566739)
Supplement: Supplementary 1 — The information of target genes in manuscript. [file 5566739.f1.docx]

Table S1 The information of target genes

| Protein | Accession Numbers | Links |
| --- | --- | --- |
| *IRF3* | B7SEE0 | https://www.uniprot.org/uniprotkb/A0A8M6Z9B8/entry |
| *C5AR1* | \| [P0C7U5](https://www.uniprot.org/uniprotkb/P0C7U5/entry) \| \| --- \| | https://www.uniprot.org/uniprotkb/P0C7U5/entry |
| *NRF2* | [Q8JIM1](https://www.uniprot.org/uniprotkb/Q8JIM1/entry) | https://www.uniprot.org/uniprotkb/Q8JIM1/entry |
| *INFβ* | [A8E6E2](https://www.uniprot.org/uniprotkb/A8E6E2/entry) | https://www.uniprot.org/uniprotkb/A8E6E2/entry |
| *PTGS2* | [Q8JH43](https://www.uniprot.org/uniprotkb/Q8JH43/entry) | https://www.uniprot.org/uniprotkb/Q8JH43/entry |
| *TRPV1* | [B6RTA2](https://www.uniprot.org/uniprotkb/B6RTA2/entry) | https://www.uniprot.org/uniprotkb/B6RTA2/entry |
| *TLR4* | [F6NLN8](https://www.uniprot.org/uniprotkb/F6NLN8/entry) | https://www.uniprot.org/uniprotkb/F6NLN8/entry |
| *TRPV4* | [A0A8M2BCH8](https://www.uniprot.org/uniprotkb/A0A8M2BCH8/entry) | https://www.uniprot.org/uniprotkb/A0A8M2BCH8/entry |
| *RELA* | [B3DHW2](https://www.uniprot.org/uniprotkb/B3DHW2/entry) | https://www.uniprot.org/uniprotkb/B3DHW2/entry |
