# Supplementary material for: Potential Targets and Signaling Mechanisms of Cinnamaldehyde Enhancing Intestinal Function and Nutritional Regulation in Fat Greenling (Hexagrammos otakii)
Source: Aquac Nutr. 2024 Apr 5;2024:5566739. doi: 10.1155/2024/5566739 (PMC11074912; doi:10.1155/2024/5566739)
Supplement: Supplementary 2 — The gene sequences of fat greenling used in the manuscript. [file 5566739.f2.docx]

**The gene sequence of the Fat greenling used for molecular docking is as follows:**

**C5**

TCTCTCTACTGACCACCAGGGGGCGACTCCTCTGGTTGTATAGAAGTCTATAGTAAATGGCTACCAGGTGATTGACAGGTCTCTACGTCTCCTCCTCAGTCCACATACGCTCACTTCCTGTTCACTGGTTTATAAAAACAAGATGGCGACGGCAAAATCACCGAACTCCAGGCTTCAAAACGTGAGAGACGTCACCATGACGACGTCCACTTCTTAAAAACAGTCTGCGTTGAGTCCAGGTCTTCACAGAGACCTGGTTCCATCGCGGTCCAGGACTCTGTGTTTACGTCATGATGTTGGAGCTCAAACAGTCAAAGATGTCGGCCATCTTATCTGCTCCTGTCTACTTGTGATGTTTGTGTGTGTGTCCCTCTCCTTCATGTGGCATGTCACCACCGTGTGACGAGTAAACCAGTGACACCAGTGATGCTTGCTGGTAGTGGAGATGAAGAGTAAACCAGTGAAACCAGTGATGCTTGCTGGTAGTGGAGATGAAGAGTAAACCAGTGACACCAGTGATGCTTGCTGGTAGTGGAGATGAAGAGTAAACCAGTGAAACCAGTGATGCTTGCTGGTAGTGGAGATGAAGAGTAAACCAGTGAAACCAGTGATGCTTGCTGGTAGTGGAGATGAAGAGTAAACAGGAAGCACCTTGAGCAGCACCGTCAGCAGCATGTACGCTGTGGTCTCCACCGTCAGGCCGCTGGATTGGTCAGGCTTCAGCCAATCAGCT

**C5aR1**

AGGCTTTTGACTAATGTGCAGGCCAGCGGCCCAAAGTGCCAGTGGTCATCATGGGCCAGAGGGATCATGAGCAGAGGGAGGGAGAGGCAGCACAGAAGATCAATCAGCGCGAGGTTGAGGAACCAGAGGGTGGTGACAGAGCGGGGCATGCAGAACCCCGTCACCCACACCACCACAGCGTTACCGGGGACACCCACCAGGAACACGAGGCCGTAGAAGACCAGAGCCACGATCTGGATCGGCTGAATCGTAGGTGTCAAATCGTCGGGAAACACAGGCAGTGTGTAATTGGTGGAATTGAAGTCTCCATAGTCTTCAAAGTAATCCATGGTTGGTTGAAGAGTTTCAGTGGCCTGTTGAAGTTTC

**MYD88**

TGTAGAATAAATTCACTTTAATAAACTGTTTTGAAAAGTCCATATCAAAAGTACAAAATCAAATGTAAAGACACAGAGACAGAGAAGGTAAGAAAGGCAGGCGTCAGCAGTCAGTGAAATTTAACAAGTGGAAGAACCTCAATCATAATATCTCTTCCTCTTAAGCTACAATCGCACCTAGAAGTTGACTCACAAGAGGAAGTGGCCAGCGCAAAGAAAAAAAAACTGAAAAAACACAGAGCATTTAGTATAGACTGCCCCAAAAACAAACACATCCTGTTCACCAATACTCCCCACATCAATACAAAACAGTGGCAGGCACAACTTGTGTTATTTTTGAAACATAAAAATGTCTTTGTGCAGTGAGAACTTTGCTGATATTTAATATCCTTGTACCTTGAGATTCATAGTGACAATATACAGAAATCACTATGTCATCTTGGAAACACAAATAGTTTCCACCATAATGTATTCTATATCGTATAAACTCAGTGTGTACTGTGCAGGACGTGGATCACAAGCAATAGCATACACTTTTAACCTGTGATGTTTGGTCATTATCCAAACCCCCTTTAATAGAACTTTAATGAATAAAAAAGGAACCTTTAAACACAATTGACTGCCTGGTTCATGGTTATGGCATTGAGAGAGCTCTCGCCAGCCGTATCCAGAACCAGGCCTGTGTGCAAGGCCGAGTGTAGTCGCATATGGTGAGGAAACGTAGGATGCTGGGGAACGGCTTCGTCATTGGCTTGTATATCACTGGAATAAGTCGTTTACTTTCAGCTCCGGGACAGAGACTGAGAGCAAACTTGGTCTGAAAGTCACAGGCGTCACTGTCGAGGTATTCATCAGAAATCACCACCACCATCTTCCTGCACCTCTTCTCAATGAGTTCACTAGTGATGCTCCACACACAGGAGCCTGGGAGGACATCTCGGTCGAACACACACAGCTTCAGCTTGTACTCGGTCTGTTCCAGCTCGCGGATCATCTCACGGACAAACTCAAAGTCCTTCTGGCAGTAGCAGATGAAGGCATCAAACGTCTCAGGAGCACCCTCGGGGTCATCATCCAGGGTGATGCCAATCCTCTCTAAGGTGCGAGAGAGACAGCTGTCCACCTCGGGGACCTGAAGTGGGGGCTTTGCATTCTTCTTCTGGTTCTCACAATACTTCCTGACATCCTCATCCATCAGAGGACGCAGATCATCCACTATGTCTCTCCTCTCCACCTCCTCCAGGATGGACAGCAGCTTCCCGACCGTCGCGTCTGAGGAGCGAGCCTGCCAGTCCTCCAAAACCGACTCGGTTGGACTTTTCGACCCCTCGTAGTTCTTTATCTCCAGGTAATTGAAGCCCATGGCCTCCGCAACAGCCATCCAGTCCGCGGCCACTGCGGTCCTGGGGTTGAGGTACAAACCCAACTTTTTACGCACGCTCATATTCAGCGCCACGGGAGGAATCGTCCACAAGTCATATTCTGAATCGACGCAAGCCATGTTGGAAAGCTTCTTTAGTTAAAACACAAACAGCGGGGACATTGTGATTTGTAAATCCGGGCGTCCACTGTCTACCTGTCAAGCGTTAAGAGGAG

**P65**

ATGGACGGTGTGTATGGATGGGGCCTGACCACACTCAACCCAGTCCAAGCGGCGAGCCCCTTCATAGAGATCATCGAGCAGCCGAAGCAGAGGGGGATGAGGTTTAGGTACAAGTGTGAGGGGCGTTCAGCCGGCAGCATCCCCGGAGAGAAGAGCAACGACACCACTAAGACACACCCAGCCATCAAGATGCACAACTACAGCGGCCCCCTGCGTGTTCGCATCTCGTTGGTGACGAAGAACGCGCCGCACAAGCCTCACCCGCACGAACTGGTCGGGAAAGACTGCAAACACGGCTACTACGAGGCGGACCTGCAGGAGAGACGAGTACACAGTTTTCAGAACCTGGGCATTCAGTGTGTCAAGAAGAAGGATGTGAATGAGGCCATCACTTGTCGGCTGCAGACCAATAATAACCCCTTCAACATTCCCGAGGCGAAGGTGTGGGAGGAGGAGTTTGACCTGAACTCGGTCCGGCTTTGCTTCCAGGCCTCCATCACTCTGGCTTCAGGGGACCTGATTCCTCTGGAGCCAGTGGTTTCGCAGCCCATCTACGACAACAGGGCCCCAAACACCGCTGAGCTGAAGATCTGCCGAGTCAACCGCAACTCTGGCAGCTGCAAAGGAGGGGATGAAATCTTTCTGCTGTGCGACAAAGTGCAAAAAGAGGACATCGAGGTGCGCTTCTTCCAGGACTCCTGGGAGGGGAAGGGCACTTTCTCCCAGGCTGACGTCCACAGGCAGGTGGCCATCGTGTTCCGCACGCCGCCATACCGCGACACTAACCTCAGCGAGCCCATCAGGGTCAAGATGCAGCTCCGCCGGCCCTCTGACCGCGAGGTCAGCGAGCCGATGGACTTCCAGTACCTGCCGGCTGACCCAGATGAATACAGGCTGAGTGAGAAGAGGAAGCGTACAGGGGACATGTTCCAGAGCCTGAAGCTGGGGCCCATGCTATCTAGTGTGTCCATGCCACAAGACAGACGGCACATAAGCCCGGCAAGGAGGACGGTCACAGCCAAGCCTCCATCAATGAATGCACAAGTTGCAGTTGTGGCGCCCCCCGGTGCCAGTGGAGCAAAAGCCCAGCCTTCCTACTCGTATCAACCGGGCCAGCTCTTCTCAGTCCAGCCGAAGGTCGAGGCCATCTCCGCTGCAACTACAAACCAGACATGGAGGATCATGGAGAGCCTGAACCTGGGCCCCCAGCCCAAAGCCACGCCGGTGGCCAACTTTACAATGAGCCAGGCAACAGCCCTCTGCTCCACTACCAGCACGTCCACCGCCAACCAGGACTACTCGACCGTCAACATGTCTGACCTTCATCAGTTCTTCCCCAACATCTCCTCGGCCATGGCCCAGGAGACGGCAGCTTCTCAGGGAAGCTCAGCCTCCTCACAGACGGGCATCTCCTTCACCCTCCCGGGCTCTCAGTTCCACGTGGATGCACCACTTGCGGACGACGATATCCCAGAGTTCCCGAGCTTTTCCGAAGCCCAGGCGCAAGGCACCCTGGAAAACCTGAACATGGACGACTTTGAGGACCTCCTGAACCCCGTCCTCATGAACGTGAGCGGAAACGGCAGCTCGATGTTGGCGCAGGCTTCGTGTCAACAGGCCGCCCCGCTCGGCTCTTCTACCGCGAGCCACAGCGCAGCGTCCCAGAACACTTCTGACCCTGCTAGCATCCCAGGAAGCACCTGGATGAATTACCCCAACAGCATCGTCAACCTGCTCCAGAACGAGGGCATGATCGACAACGGCAACCACCGGCCCCCGGTGCTGGACGAGTTCGACGAGTTGATGTCCGCCGACGAGGACCGTCTTATTTCCATCTTTAACAGTGGAAGCCAAGCTGGGTTCGTTTCAGGACACCCGACCTAA

**IL-6**

AACCACACAGCACAAGCTTTATTCACACTCTCCATAGAGAATATTTGAACGTTAGGGACATTGCAGAAGTAGGTTACGGTTCAGGTTTATTGGCAGTTCGATGCTCTCTATTATTGAAGAAAAAATATTTGCAAATGTGTGCAACTGTATTGCAATCCGTGTGTACGTAATGACATTTGTAAATGTACGAGAGAATCTCTCAATACATACTGAGATTTGAGAATGTATAAAAAAGAATCTGCAAATGTATAAAAAAAAAAATCTGTGTGTGCTGTAGTAACTGATTACTTACAGCTAATATCTATTTACACATTTGTCTACAGGTTTACAAATCTCAATATACTTGCAAATCTGATCATTTACTAATCTCCTTAGATATTACATGAATACACTTTTGCAAATTCCTCTGCACATTCACAAATATCAGTATGAATTTAAAGATTATTTTACACATTTACTTCTCTGATTACACACACGATTTGAGACACAGTTACCCAACTCTCCAAACACATCTGCAAATAGTGTTGCTTCAATCATGGCCCCATACTATTTTCATTTTACCTTTAATCACAATCATTAGAAATGTCCGTTACTGACAATAAAGTTATTAACTGAACAATTAGTGGACTGAAGCAAATGTATTAGTGTCTTTGTTGTTTCTAAAATAGAGTCCATCCGTGTGTTGGTTTGTTTCACTCCATGTTTCCTTCCATTTTGGAGCCAATAACCACATGAAGATCTGGATTGGAGAAGTGATCTGCAGCAAACTCATGGATGTCAGCGGGCCTTTTACGAAGCACATCTCCCAAAAAGTCCCCCATCAACACCTCCACCTCTGGATGCGACCTCAAATACTTCTCGTTGTCCATTCTCGTTTTGATCTGACAATCACAAGAAACAAAATGCATATTAAGGGTTTATAATGAGCTGCACGCGAGCTGCAGGGTGTCGCGGTGAGTTTGTACTGCGTCACCTTGAACTGTCGCAGCTTCTCCTGCTGCTCAGCGCTCAACACACCCACGTCCACTTTCAACAAACCTCCATTTCCAGCCATGATGTCTGTTCGTGTAATTATTTATGTATTAACGCACAAACACGGTCACCTGCTCGTGACACAGCTGCTGCGGGCTGGAAACATATGCTGCTATGGAAACGACCTTCAGTAATTAAGGGCTGCGTCCTCGAGGTGGCAGCACGTGACAGGGAAAGAGTCCCCGGTGTATTCTTGTATTGTTATTTCAATGAATCCACATGTTTAAAGCAGGAATAATTTGAGATATACATGATAGGTAAACAGCAGTGTTTTGTCTGCTAAGAGTCTCTTAAAAAACCTCAACTTTTTTTATTTTTTCTGTAATATTGCAAGAACAGAAAACCAGCATGGTGGTTGGAGTAAAGTTTCATCGCTGACTCAGTTCATCAAATTCCACACACCAAAATACTATTTGATAAGGTTTTGTGATAAGATGCATTTCTTTCTGGGAGTGACTGCTTAGATTTAGTTATGTAGTCACTGAAAACTATTTTTTTCATTTTGTATCCCAACTCAATATGCTCAAAATTAATGAGTGCAGTTTACCTATGCTTTCTACACTAGAATAATGGGCATCATGGCACCCGGTTTGCTAATTTGATTTTATTAAATTAATAACACAAAACTAAGGTAATGGAGGTCTTGAAACTATATTGCATTTCCTCTTCAAGACAGAGGCTCAAGATCAAGTAATAAAGCAAGACCCAACTTAAGGCCTGTGTCATATAAATTAATATTATTCTTACGAGGTCCTAGAGCCTGGAGTTCAGGGGCCAACACAGAACCATAAACAAGACAATCAGCCCGGCCTAATCAGTCTAAAGGACGACAACCGATGAACAACCAGTGCTTTTGTGAGAAAGGCAATTTGGCAATTCTTTGTCAAACCTTGTAAAGAATATGTTTAAACTGAAAGGGCAGGCAGCAAGTGATATATTTGATAAACCTTAATAGAAATGACATACAAACAGAACATGAGGTGATATGCTTGACGCAGACCTATGATGAGGCCTACATCTAGTCTACAGTTTATCAACACACTTCAACTCAGCACAGACATGAGAAAGAGTAGCTAAAAGGCTGCTCTTGAGACAAATACAATGACAAAAAGTCTTTGAGAGGTATTTTAAGCATTAGTTAAAGATACAGTAAAAAGAGAGACAGATTAAAAGCAGTTCCTGAGTTTGCTTCCAACAGAAGCTCATTGGAAAACAATAACAATTTGAAGTCCAATATCCTTTAGTCATCTGTGACATTGGTTAGTTGTATTCCCTTTTCTGCCTGCTCATCAGTCCTTTGGAGGAAGAAATAACTTGTGTTGTTGAAGTGCATGGCTTCTACCCTCTCCTTAGGCCGCTGTTCTTCATTTTCTTCTTCCCCTTCTTCCACTTCACTCTGTGGGGTTTCTTTGTAATGAGGAGGGGCAAAGGTCACCAGAGCCTGTCCTTCTGGAGGTGTTAGGGTGGGAGGCGGAGGCCGTGGAGAGTCCCCACACTGTATGACGACACTCAGACTCTGGAGTTTAGACATAAATCTGTCCTTGTGTCTGAATATGTAGGCAGCCAAAATGACAAGAGACAAGAGAGCAAAGGAACCAGAGATGCACAGGACATGACGTAACACCTCCACACCACTTTGGACCGGTTCACGAACATCAAACGGATCATAATCTGTACCAGAGCTGCCTTCGTTGTCTGTGTATACTGGAAACGTTGTGGCAGAGAGATCATCCTCCGACAACTCATTTGACCCTTTAGCTGAGGAGAGAGAAATCCATGCAGATGACATGTTATCAACAACTTTAACAGGAAGGAAGCTCCTTCATCTAGACAATAAAAAATGTATGAAAAGTAAAAACATACTGTATGGTAGATAGTGCATTGTTTAGACCAGTGTTTCCCAACTGGAGGTTGGGAAGAGGTCGCAAGAAAGACCAGCAAGACAGGAACAAATAAAATATTTCTGCTACACCAAATAGTCTTTCATCTCATCACAAGCAAAAAAGGTTGGGAGCCACTGATCTAGATGGAGTTATGTTATTGTATTATTCTTTATTAGTAATTATATAGTCACGTTTTGTTTAAAACATCATTACCTTTGGATTCAAGACTCATTAAAGCATTACATTTTTTGATCTACAGACGTTTTTTAGAGTGAAAAGTTATTTAAAGAGAAAAAAAACTCATACAAACAAAATATTCAATATTTTCTAGTATGAAATACTGTATGTATTTTTTGTCCTGTAAGTTTTTTGTCCTAACCTGCCCGACAGTATTATACATTGACCCAGAATGCTGCATGCATTCATGCTGTGCACAATGACAACTCATTCAGAGAGGAAGAGAAGCACAATCGGGAGGGCTAAAAGTAAGTACATATGGGAGGGTGATTATGAACAGCTTATTGGGAAAACCCAATGATACGGGAAGGAAACAGGAGGCCTTCCTCCTCTGGTCGTCATGGCTACATTTGTCTGCCTCCAGGGTGGATCATGTATTACAAAGATGATAAAGCTCAATGATTTACAGGTCCCCGTAATCGACGTCACTTCCATGTCTCGAAAACCAATAACATACTTTTTCTGCCTGCTGGCTCTTGAACAAGAAAGAAAATACTAATGTGAAGTTTCCACGAACCATAGCTTCAACTAAATGCATGTCCCAATTTTTAAATGGCTTGATTTGATACATTTCTTTCCACATGTATGAGTGTGTGCATGTGTGCTTACTTGTTGTCAACAACCAACTGCTGGCATAGACAGGTGTACTCCAGTCACTCCACAGACCATCATACTCTTCCCTGGTTCTGAGTTGTATCGAGTACTCAACACCAGGCATTGCATCAGTGATTGTGTAGGAGCGCTCCCTCATCGTCTGTATCTGGCCGTGATATGACGAGGACTTGAGAGGTCGGTATTTCATCTCGTAGATTAGTACATAATAGCTGTCTTGAGACTTCCAGGAGTTGGGCAAACTCCAGGTAACGGTCATCCTCATCTCCTGTCCCTCCTCCTGTCGAACTGTCACTTCTGATGGAGGGTCAGGCTTTACTGAGGGAGTAAGTAGGAATATAGTAAGTGCTATCCATCAGACCAAACACACACGTGACATGGACACACACCCGCCTCGACACACAAACGTACAAATGTTCAGAGGTAGGAAGTGCAACAGGGTGCTAGTGGCGTTTCCTATGATGCTCGTAACACACAGGTAGGCCATGTGAAGGGTTCTCTGCTCGTCCTCGTTGTGGTCCAGAGCACACCAACAGCGGGAGCGTCGATATGAGTATGAGCACTGCGATCGAAGGAATGCCTCCGTCGGGCTTTTACTGAGTAAGAGATAACAGTTAGGCCCTTTAGTGATGGGCTTCTGAGGTGCCCATTCACAGCGAATCTTACTGCTGGGTGACCTTTTGTAGCAGGACAGGACGGGCTTCTCTGGAGGATCTGCAACATTTAGTTTTAAAGAGAACCTCTCTCTGCCTCTGTAATGACAAGTGTACCTCCCGGAGTCCGTCAGTCGTACCGAGGACAGAGACAGTGCAGCCCCTCTCCTCTCAAATGTGATTTCACCCCAGTCTCTGTCTTCTTTCCCCACCGTCCTACCGTTCCACTTCCACTGGAGCCTTGACTTTATGCCCCTTGTTACTCTGCTCCCTTCCTCCGCTCCTTCCTCTTCCTCATAATCGTCCATGCCATCCATCTCCTCAGCCTCCCAGTCGGATTCACCCTTTGATTGTCTGCTTACACTGGTTGGCCGGACTGTGTGAGGGGTGGCAGAAGCTGTGTATCCTGTATCTGTAAGGCTTCTGCTTTCTCCAGCCGAAGTTGAGTTAGACTGGTATCCCTCATTTACAGTATTCTTCAAAGTATGTTTTTCGCTTTTCATCCAAACTCCAGTTTTGCTTACAATGTTTACAGTGGTTGGGGTTGGTGTTGCATGAGAAGAACTCCCACTTCTGTTGTTTGAACTGTTTCTGTGAATGCTGACCTTCACTCCGTCCACCTCCACATGGCCACTGCAGGTCAAAACTAACTTACTGCCCGGGGATACAACCAACCAATCAGGGGGAGGGTCTTTTCTGGGACAGGTTCCGTCGTAAATGCCCCGGACGCAGAGAACAAACAGCAAAGGGAGAAAAATCCACATGTTTGCACATGAGTCTCCCTCACCGGGTCCTCACACCATCACCTACACACAAACAGCACCAGATGGAGATTGTCCTGGCTCTAATCTCTTCACACGAACAACAACAACAACTGAGAATCCTCCTCTGCGGACGGTTGTTGAAGCAGTTACCGGCTTCCTCTGGTTGCAGCAGTCTGCTGTCAGTAAG

**TNF-α**

TCAGCTCGGGTTTTGACCACAACCGGCCCAGCTGACCAGTTCATCCTCTCGCCCCGCCACTATCAGCACACCGACCCCGACTCGGGCACCCAGCTGGTCTGTGATAAGTGCCCGGCAGGCACCCACGTGTCCGTCCACTGCACCCAGAAGAGCGTGAGGGAGTGCAGCCGCTGCCCTGACGGCACCTTCACGCGGGGCGAGAACGGCATGCAGAAATGCCATCCCTGTCGAACTCCGTGTCCTGCAGGCTTTTTTGAGAAAGCCATCTGCACGCCCACCCAGGACCGAGTCTGCACGTGTCCCCCCAACAGCTTTTTGGTGGGGGATGGTGGCACAGAGTGTAAGCCGCACTCGCTGTGCCCGCCAGCGACCAGGGTGAAAAGGCGGGGCAGCGAAACAGAGGACGTGCTCTGTAAGCCGTGCACCAAGGGGACTTTCTCAGATGTGGAGTCGAGCGTGGTGAAGTGCCCGACCCACACGGACTGCCAAGCTCAGGGGCTGGTGCTGCTCACACCGGGGACTAAAGAGACAGATAATATCTGTGGAGCCCCTTCCGCAGCCCTCGCTTCCTCTTCATCTCCGCCTGTCTCCACTACCACCCTGCTGGGGCCGGCACAGGCTGTTCTTGTACTGGAACCCATGCCTTCTTCATCCACTCCATCATCATCCCTGACTGGACCTGCACATAAAGGCACCTTCAGCCAGTCCGCAGCCATTCAACTGAGGGGAAACTCGGGTGGGGAAGACCACCATGCAGAGGGGCTACTGCTCAGCCGGTCTGGCACTCCCACCCCACCAAATCGCGATCCACCACGGGGTTCACAGCTCCCAGCTCACAAGCAACTCATGGACCTCGAGCAACCACGTGGGCAACCCAGTTCTGACCCTGTGCCAGGCCCCAACAAGAGGCCGGTGGAAGGTCTGGAGAGTGTAGAGGTGGTCAGGGTTGGTGTTAGCAAGGTAGCCGGGCAGGGGAGCGGAGGTGGTGGAAGTGGGGTCTCCAGCTATTACAGGCCCACTCGCAGGGGGTCCCCAAGGCCGAGCACCCATGATCACTTTGACATCAACGAGCACCTCCCGTGGATGATAGTGCTGCTGCTCCTGCTGGTGCTGGTGGTGATTGTGATCTGCAGCGTGAAGCGGAGCTCTCGCGTGCTGAAGAAGGGCCCCGTGCAGGACCCGAGCAGCATCATCGAGAAGGCCATCCAGAAGAAAGCAACTACACCTCTAACCCAGGTCAAAGAGAAGTGGATCTACTACTCCAACGGACAGGGGGTGGACATCCTGAAGCTGGTGGCGGCCCAGGTGGGCACCCAGTGGATCGACATCTACCAGTCGCTGGCCAACGCCACGGAGCGGGAGGTGGCCGCCTTCTCAAACGGCTACTCGTCGGATCACGAGCGGGCGTACGCCGCGCTGCAGCACTGGACCATCCGGGACGGCGACGCCAACCTGGCCAAGCTGATCAACGCCCTGCACAGACAGCGGCGCATCGACGTGGTGGAGAAGATCCGCTGCGTCATGGAGGACAACCCGCAGTTTGACATCAACCAGCTGATGACCTCAGTGAATGTAAGCCAAAGCATCAGTCCCACCCACAAGCCGCTCGAGTCTCCCAGCAGCGTCGGCAGCAGCAGCAACGGCGGCGGTAGCGTTGGCGGTAGCGTTGGCGGTGCCAGCGTTGGCGGTGCCAGTGTCATAGGGGTGGAGCCGTCGCCGGTGGACCGCACCAAGGGCTTCTTCCCCGACGAATCGGAGCCCCTGCTTCGCTGTGACTCCACCTCCAGCAAAGACTCCGCCCTCAGCAGGAACGGCTCCTTCATTACCAAAGAGAAGAAAGACACCGTGCTCCGGCAGGTGCGTCTGGACCCGTGCGACCTGCAGCCCATCTTCGACGACATGCTGCACATCCTGAACCCGGAGGAGCTGCACGTCATCGAGGAGATCCCGGCCGCCGAGGACCGGCTGGACCAGCTGTTCGAGATCGCGGGGGTCAAGAGTCAGGAGGCCAGCCAGACACTGCTGGACTCGGTCTACAGCCACCTCCCTGACCTGTTGTAGTGCAAGGCAGGAGGGAGAAACCACGCGGATGAGAATCGGCACAGGAGTAAAACAAGGAGTGTCTCTGTGGGAGGGATGACTCAGATCGGAGTCGCACATGTCACACTGTCACATGATTTGATCTAGGTGTTGTAGATGAGGTGTGATCAATTCCCCTCAAATGACTCTTTCAAATCTTACTGGTACTTTACTCCTCTTTACCCTCTTCAGTTGTCTTGTGTGTACAAGCTATTCCTTCAAAACACCTCCACCCTCCACCAAGATACAGGGTCAGCGCTCAATGGGATTAAAGCACACTGAGTGTCACACCAGGAAGTACTTATGTACTACGTGTACAGCCTTGTTTAAGGTGGTATTATGAGAGTATTTTGACCAGAACAATGAAGCTTAAGAGCTTCCAAACTTAGTTTCTATATCTCAAGTGGCCAATGCCTTCACCTTTAGAGGGTTTTACAGGTGTTTTTTGTCGGCCGTGCCAATCGTCCTGGCTTCCCCTGCCTTTAACATGATTTCAGTGTTTAAACAAAAACAATAAACTTATTTAACATGGTGGTTATTGCTAAATGATTGCAAGAAAGGAAACTACACACAGACAATAGTAAATAAGCTATAAATCTAGATGACACTGGTATTATTCGTAGGGTTTTAAGGGGTTTAGGAGTTTTGTAAACCACATTCACAGATATTTTGCCCAGGTATGATTTGGAGGAGCTGGTTCCCTTTCTCAGCCCCTCATGTTTTTTTCTACCACGTCTAGTTGCCTGTAACTGGAGCAAGATCATCTGTGGGTCATTTTAACTACCAGACCAGCAGGGGGCAGTGTCAGATTCGAGGGCTGACCTGCTGCTCTGGAACCAAAGCCATATCTCAAATAAATACTGGATTAAACGTCAAAGTAATCCAATATTCCATTAGTGGAGTAATATTCCCCTAATCTAATATAATGATTTATGGCTTTGCCTTTGAACTTTATGCTTTTGGACGAAAGGTTCTATAACTATCAATGAACATGAAGGGAGCAGTATTTACCGCCACTGCCTTTGCTTCTCTGGAACCAAAGTCGTATCTAAAAACGGTTTTAGATACGTCGCTTTGGAAGCTCTGTCGCTGTTGGACTCAAGGACATTATAGAATTCATACTTCTACCAGTACGCACATCCAGATCTCTGCTAGCTAGCCCCGTTTGCCTCAACAAATGTAGCGTTGATAAAGTGTTCTTGATACATTTCGGAGCTTCGTGTATGGCTGTCTGAGTGTTTCAGTGCTACAGTAAAAACAATAGAACACAAGTGATTTTTTCTTCTTCATGCGGTGATATGTTGCTCTAACAATGATGCACTAATTCTATTTTCTAGAGGAGAGGCTGACTGTATTTTTTCGTGTCCTTCATTTTCACTGTCTTTAATTCTGAAATGCCGTTGCCTAACATTTTAATTTTGTTATGTTTTCTACATTTAGTTTGATTGCGTCATTGTAGGACCTCTTTGTTATAATCCTGACTTCATTGTATCTGGTGTTTTCGCTGGCATTCAGATGTATTCCCCCCCACCACCACCACCACCGCTTTGCTTGCTGGTGATGACCTGGCCGGGATGTAAGTTATGCATTACTCCATACTTGAAGTCTACTTGTGTTACTTGATTGAAATGAGGTAGAAATAATGTGCTCTTTCAGTTCCTTCCAAGTTCCAAGTTCTTAAAAGAAATAATAAAACTGAAAAGCACCACCAAAAAAAAACTCATCGACTTTGGGAGAGGACTTTGCAGCAGGCGCCATGAGAGCAGCAAATGATGGAGTCTGTTTTCACCAAGCCAGTTACTATCAAAGCTCCCGTGGTGCTCGATGTGAACTCACCGTTGGCCTGACGCAGGGGTCTAACCCCGGACTAGTGACTACTTTAAGTATTCCAGACAGAGCGAAGGTTGTGTTGCTGAGGATTGTGGGATGTTGACAATGATGGGGATGAGAATGTGTGAGCTTGCGCTCCACAATGCTGTGGAGACTCAAACTGCAGGGTGTGAGCTTTTTCTTTTCTTTTTTTTCTTATCTTCTAAGGAAGTCTGACAGGATGATGGTGAGGTGTGAAACACACATGTGGCACGAGTGGATGATTTCATTAATGTGTGTAACCAGTATGTTTACTACTATGTAACCTCTTAAATTCAACCAATTAAAGCTCAAAG

**PTGS2**

CTGAGAAAGGTGATCCATTTCCTTGGAGCATTTATTAGTGTCAACTTTGCAAGTCAAAAGGCATTGAGAACAACATTTACAAACTGGTTATATACAGTACATGGGTATTTTAAGTCCATATAACTGAATGAACTGATGTATACTATGACAATGCAATTCTGAACAACAGTACAGTTAAGTTACTAATGCCATTTCAAAGTACCCAAAGCCAGTACGCAGACTGCAGTAGAAAAACAATCAGTAAAACAATATTGAAGTAGGAACATTGGATCAAGTGTGTCAGTCATTAATTTCACTGTGAACATTACTACTGTACCTTTGTAAAACATTGGGACAACTGTACAATTTCATGAACATAAATGGATGAGTAGCAGTTGATTTAAGGGAACATTAGGAATGTAAAGTACGCTACTATGTAGTTGAAACTGTAAACTAATATTCAAGTATCAAGTTTTTTAGTGTCTGAGGTCTTTACTGTAACAATAATTTATAATACAATGAATCTTTCAATAAATAAACACAACCACAGACTTTTGTTTTTGAAAATCAGACAGAAATATATAAAAATACATATTAAAAAATGTTGTGTCATAAATAGAATACAATAAATACAAAAATAAATAGATAAATATTAAAAATATTCGTACAAAATAAAAATAAATTAGAGCTCATTAGTCCTTTCTTTCAAAATGACTGTGGGGTTGATATCACCGTTGCGCGAGTGGGAAGTGCTTGAGTTGATGATCATCGACCCTGTGTCTTTAACGTTGGGTACAGAAAAGGATGCCACGGGACAGGGGCCTTGGACGTTATTGCACACGAGCCTCTGCAGGGAGGCGGTGTTGACGATGTTGAAGCCCACGCTGCCTCCGAATGTGCTCGGCTTCCAGTACTCCGGGGAGCAGATGGGGTTTCCCATAAGGCCCTTGAGGGAGTAAGGGGCCCCCATCTCCACCATGGTCTCCCCAAAGATTGCGTTAGACCTGGGCTTCTCCACCAACAGACCTGGGTAGAGCTCCACAGCGTCGACGTGTCCGTAGAACTCCTCAAGTACTGCAGCCATTTCTTTCTCTCCTGTCATGTCTTCAAAAGAGCTGTAGGGCTTCATGGAGAAGCGTTTCCTGTAGGCGTTCAGAGACTGGTAGCGCATTTGTCTGCTGTTTTCAATGGACTTAATGGCCACGTACATGATAGGTCCTGGCACATTTTGCCCGCCGGCAACCCGTCCAGCAATCTGATTGGTAAACGATTCCACGAGGTTGCCGATGCCGTGCTCGGTGACTACGGAGGTGTTGAAGACAAACTCTTTATAGCTGTAATCTTTCTCCTCAATGTGGAAACTGTCAGGCATCAGAGGGTGCCAGTGGTACAAGGTGTTGAACTCGGATGCAATGCGATTCTGGTACTGGAAGCGCTGGTTGAACAGCAGCTCGGGATCAAACTTGAGTTTGAAGTGATAACCGCTCAGGTGCTGCACGTAGTCCTCGATCACAATCTTGATGGTCTCTCCAATCAGAATTAGTCGTGTGGTCTGGAAGAGCCTTTCATCGTCCCAGTCCGGGTGGACCTCCTGCAACACATCACACACTCGGTTGTGTTCTCGCAGCCAGATGGTGGCGTACATCATCAGACCGGGGACTAGGCCGAATGCCTCATGGCCCACAGCGAAGCGGTGAGACTCAGGAACGTGAGGAGGGTAGTGCATGTCAGCGCCTACTTCCTTTACTGTTGGGGGGTACATCTCTCCATCCAGGATCTGATATTTAAGCTTGCCATCCTTTAAGAGTCTGAGCTTGTATTGCCTCTCCAGGCCGTCTCCATAAATGTGGCTGAGGTCCACCCCGTGTCCTTTAGCTAAAGTAAAAGCAGGTCCTTTCTTCATATCAGATTTGAAGAACTGGTGGGTGAAATGCTGTGCGAAGAATGCAAACATCAGGCTGGTGCCCTGTGGGTCCGGAATGAACTGTCTTCTCATTAGAAGCTTCTCAGCCAATAGCTTAGCATCAGGCAGCTCCTTTTTACCTACTACTCCCATAGGGGTTGGGCAATCCTCTGGCACAGGGGGGAGGGTGCGGGTATAGTAGGAGAGGTTGGAATATGCTTCCCAGCTTTTGTAACCATAATCTGCATTGAAGGTCGGAGGACTATCAATTAAGTGGGATCGAGATGTCAGCACATATGTCATAATGGCATTCCTGAAGAAGGAGATGTTGCTGACGATGTTCCAGAAGCCCTTGAAGTGGGTGAGCAGGTAGTGGACAGTGTTGGGCGATGGCTTCAGGGATATTTTGATCCAGGTGAGGAATTCAGGCGTTGTGCAGTTGTGTCCATGGTATCCTGTGCGTGTGCAATCACACTCGTAATTATCAGTTCCAAGTGCTGTGCAAACGCCCCTGTTCTGGCATGGCTCTGAGCAACATGGGTTACCTTCACAGACCAGAACACCCAGTGCCAAGAGGAAAACCGCGAAAGTAAATCTGTACATAGTCCAAAGACTTTGTCGGATCTGGTTTTTCTTTCCTTCTCTCTC
