# Supplementary material for: Potential Targets and Signaling Mechanisms of Cinnamaldehyde Enhancing Intestinal Function and Nutritional Regulation in Fat Greenling (Hexagrammos otakii)
Source: Aquac Nutr. 2024 Apr 5;2024:5566739. doi: 10.1155/2024/5566739 (PMC11074912; doi:10.1155/2024/5566739)
Supplement: Supplementary 3 — The protein sequences of fat greenling used in the manuscript. [file 5566739.f3.docx]

The amino acid sequence of the Fat greenling used for molecular docking is as follows:

＞C5AR1

MDYFEDYGDFNSTNYTLPVFPDDLTPTIQPIQIVALVFYGLVFLVGVPGNAVVVWVTGFCMPRSVTTLWFLNLALIDLLCCLSLPLLMIPLAHDDHWHFGPLACTLVKS

＞PTGS2

MYRFTFAVFLLALGVLVCEGNPCCSEPCQNRGVCTALGTDNYECDCTRTGYHGHNCTTPEFLTWIKISLKPSPNTVHYLLTHFKGFWNIVSNISFFRNAIMTYVLTSRSHLIDSPPTFNADYGYKSWEAYSNLSYYTRTLPPVPEDCPTPMGVVGKKELPDAKLLAEKLLMRRQFIPDPQGTSLMFAFFAQHFTHQFFKSDMKKGPAFTLAKGHGVDLSHIYGDGLERQYKLRLLKDGKLKYQILDGEMYPPTVKEVGADMHYPPHVPESHRFAVGHEAFGLVPGLMMYATIWLREHNRVCDVLQEVHPDWDDERLFQTTRLILIGETIKIVIEDYVQHLSGYHFKLKFDPELLFNQRFQYQNRIASEFNTLYHWHPLMPDSFHIEEKDYSYKEFVFNTSVVTEHGIGNLVESFTNQIAGRVAGGQNVPGPIMYVAIKSIENSRQMRYQSLNAYRKRFSMKPYSSFEDMTGEKEMAAVLEEFYGHVDAVELYPGLLVEKPRSNAIFGETMVEMGAPYSLKGLMGNPICSPEYWKPSTFGGSVGFNIVNTASLQRLVCNNVQGPCPVASFSVPNVKDTGSMIINSSTSHSRNGDINPTVILKERTNEL

＞TLR4

MARALAGDRMVRELGREYDGKPIVQNITDAIYGIRKTICVISRHYLQSEWCSREIQMASYRLFDEQDDVLILLLMEDIPVGELSLYYRMRSLVKRRTYLSWPHDPAVHGGHPCPRAVSVLPNEESGEETHLPELAASRSTHRSLLAEHTASSDGTWKSHGQPNQGFLSEPGMKSNL

＞P65

MDGVYGWGLTTLNPVQAASPFIEIIEQPKQRGMRFRYKCEGRSAGSIPGEKSNDTTKTHPAIKMHNYSGPLRVRISLVTKNAPHKPHPHELVGKDCKHGYYEADLQERRVHSFQNLGIQCVKKKDVNEAITCRLQTNNNPFNIPEAKVWEEEFDLNSVRLCFQASITLASGDLIPLEPVVSQPIYDNRAPNTAELKICRVNRNSGSCKGGDEIFLLCDKVQKEDIEVRFFQDSWEGKGTFSQADVHRQVAIVFRTPPYRDTNLSEPIRVKMQLRRPSDREVSEPMDFQYLPADPDEYRLSEKRKRTGDMFQSLKLGPMLSSVSMPQDRRHISPARRTVTAKPPSMNAQVAVVAPPGASGAKAQPSYSYQPGQLFSVQPKVEAISAATTNQTWRIMESLNLGPQPKATPVANFTMSQATALCSTTSTSTANQDYSTVNMSDLHQFFPNISSAMAQETAASQGSSASSQTGISFTLPGSQFHVDAPLADDDIPEFPSFSEAQAQGTLENLNMDDFEDLLNPVLMNVSGNGSSMLAQASCQQAAPLGSSTASHSAASQNTSDPASIPGSTWMNYPNSIVNLLQNEGMIDNGNHRPPVLDEFDELMSADEDRLISIFNSGSQAGFVSGHPT*
