# Supplementary material for: Potential Targets and Signaling Mechanisms of Cinnamaldehyde Enhancing Intestinal Function and Nutritional Regulation in Fat Greenling (Hexagrammos otakii)
Source: Aquac Nutr. 2024 Apr 5;2024:5566739. doi: 10.1155/2024/5566739 (PMC11074912; doi:10.1155/2024/5566739)
Supplement: Supplementary 4 — The percent weight gain and feeding rate of H.otakii for feeding 8-week. [file 5566739.f4.docx]

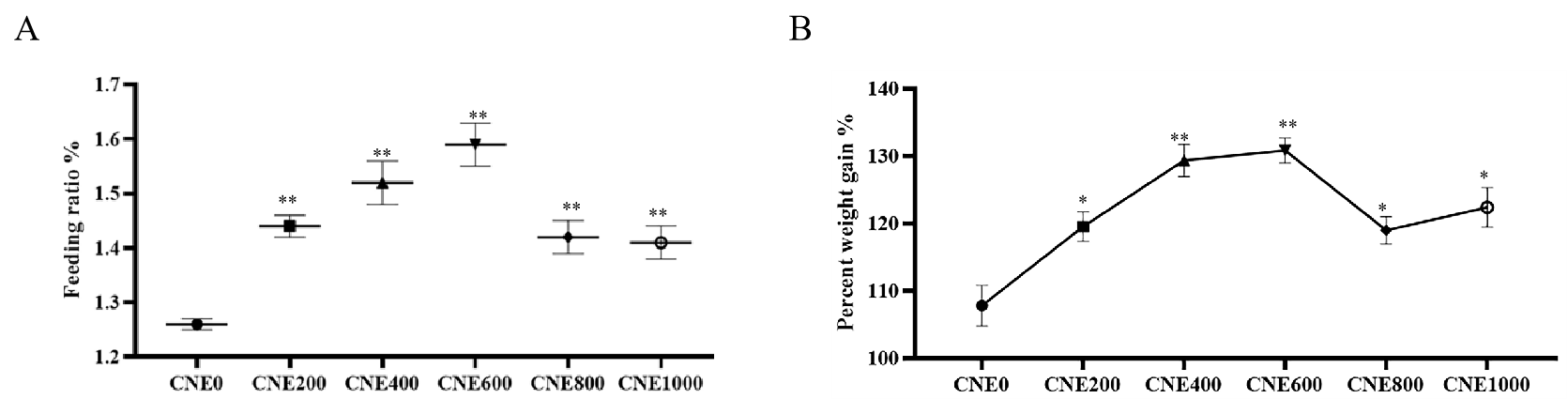


**Figure S1.** The effect of dietary supplementation of cinnamaldehyde (CNE) on percent weight gain and feeding ratio in *H.otakii*. (A) Feeding ratio; (B) Percent weight gain. The identical indexes with * and ** had obviously different mean values (*P* < 0.05, *P* < 0.01), and the “ns” denotes that there were no remarkable differences between groups.
